# Special Issue “Plant Extracts: Biological and Pharmacological Activity”

**DOI:** 10.3390/molecules25215131

**Published:** 2020-11-04

**Authors:** Raffaele Capasso, Lorenzo Di Cesare Mannelli

**Affiliations:** 1 Department of Agricultural Sciences, University of Naples Federico II, 80055 Portici, Naples, Italy; 2 Department of Neuroscience, Psychology, Drug Research and Child Health-Neurofarba-Section of Pharmacology and Toxicology, University of Florence, 50139 Florence, Italy

The use of plant extracts for therapeutic purposes knows a wide diffusion. The vegetal origin of these products intercepts people’s desire to cure themselves with natural drugs; this aspect, together with effectiveness and regulatory opportunities, is the base of the modern broad use of medicinal plants. Traditional uses and novel biological effects allow for availability of an extraordinary high number of different compounds that may constitute a formidable therapeutic potential. Nevertheless, pitfalls are hidden behind poor pharmacological and toxicological knowledge of plant extracts, non-standardized methods of extraction, and undefined and not repeatable qualitative and quantitative composition.

In this context, novel experimental studies on plant products are necessary and appreciated to reinforce the scientific soundness of phytotherapy. This Special Issue aims to respond to this medical need, comprehensively highlighting the newest discoveries in vegetal resources with an emphasis on pharmacological activity. More than 30, highly cited, articles were collected.

Cheng et al. [1] showed the hypoglycemic and anti-dyslipidemic effect of purified anthraquinone-glycoside from *Rheum palmatum* L. in a rat model of type 2 diabetes mellitus. The anthraquinone can reduce oxidative stress and regulate Fas/FasL-mediated apoptosis signaling pathway improving β-cell function.

The dihydrochalcone 3′-*O*-β-d-glucopyranosyl α,4,2′,4′,6′-pentahydroxy–dihydrochalcone isolated from *Eysenhardtia polystachya* was able to protect mice against diabetic nephropathy. It improved renal dysfunction as well as it reduced glycated hemoglobin and advanced glycation end products in the presence of significant histological recovery of kidney [2].

Gunathilaka and coworkers [3] reported the hypoglycemic and antioxidant in vitro activities of the marine red algae *Gracilaria edulis*. A de-polysaccharide methanol extract of *G. edulis* was sequentially partitioned by different solvents. The ethyl acetate fraction exhibited the strongest hypoglycemic and antiglycation potential. Gas chromatography-mass spectrometry analysis of the ethyl acetate fraction revealed the presence of several candidate anti-diabetic compounds.

Chrysophanol and physcion, isolated from the root of *Rumex crispus* L. emerged as relevant in the medicinal properties of the plant against gout and diabetes. Compounds showed scavenging capacity, xanthine oxidase and α-glucosidase inhibitory activity [4].

Quan et al. [5] showed the result of a phytochemical and pharmacological study on the *Canarium tramdenum* bark. Five different extracts were chemically characterized, revealing that *C. tramdenum* fruit possesses phenols and terpenoids, which might contribute to reducing risks from diabetes. A high quantity of α- and β-amyrins highlighted the potentials of anti-inflammatory, anti-ulcer, anti-hyperlipidemic, anti-tumor, and hepatoprotective properties of *C. tramdenum* bark.

An ethanol extract of *Croton hypoleucus* showed antioxidant and hepatoprotective activity in rats with liver necrosis. Hepatic damage markers were reduced, whereas *SOD* and *Cat* gene expression were increased, suggesting a control of antioxidant defense levels [6].

Recinella et al. [7] described the antinflammatory effects of polyphenolic liquid complexes from olive pressing juice with high levels of hydroxytyrosol. In isolated rat colon, liver, heart, and prefrontal cortex samples, a tissue-dedicated molecular analysis revealed that the products exhibited protective effects on multiple inflammatory and oxidative stress pathways.

The Korean plant *Aucklandia lappa* Decne., known as “Mok-hyang” was investigated as ethanol extract for studying in vitro the anti-inflammatory and antioxidative effects. The extract reduced redox unbalance and proinflammatory mediators by decreasing the nuclear translocation of p65 and by the enhanced expression of hemeoxygenase 1 [8].

Nwokocha and collegues [9] reported the cardioprotective effects of the juice of *Melicoccus bijugatus* fruit pulp. Using a rat model of myocardial injury, the authors showed that a repeated treatment reduced blood pressure and heart rate as well as decreased heart to body weight ratio. Vitamin C and related compounds, phenolic acids, flavonoid, fatty acids and terpene derivatives were individuated as components.

In vivo and in vitro experiments, as well as a UHPLC-Q/TOF-MS-based metabolomics study, showed the effect of the dry rhizome of *Rehmannia glutinosa* Libosch. in preventing osteoporosis and its underlying mechanisms. *Rehmannia glutinosa* enhanced bone mineral density and improved the micro-architecture of trabecular bone via interfering with the steroid hormone biosynthesis [10].

Regarding the nervous system, Silvestro et al. [11] studied the effect of cannabidiol, one of the cannabinoids with non-psychotropic action extracted from *Cannabis sativa,* against the severe treatment-resistant epilepsy in addition to common anti-epileptic drugs. An overview of recent literature and clinical trials showed that the use of cannabidiol could represent hope for patients who are resistant to conventional anti-epileptic drugs.

Cannabidiol was also studied for toxicological aspects [12]. In mice, cannabidiol induced signs of hepatotoxicity, possibly of a cholestatic nature; hepatotoxicity gene expression arrays revealed that it differentially regulated more than 50 genes, many of which were linked to oxidative stress responses, lipid metabolism pathways and drug metabolizing enzymes.

Tan and coworkers [13] described the anti-inflammatory and neuroprotective properties of *Guettarda speciosa* (chloroform and methanol extracts)*. G. speciosa* was able to inhibit cyclooxygenase assay (partial selectivity for COX-1), further it reduced amyloid-beta aggregates in the neuronal cell line, suggesting possible anti-neurodegenerative applications.

Beta-amyloid-induced neurotoxicity was also prevented by an aqueous extract of *Morus nigra* ‘Chiang Mai’ [14]. High amounts of cyanidin, keracyanin, and kuromanin as anthocyanidin and anthocyanins were found in the extract. *M. nigra* promoted neurite outgrowth and improved locomotory coordination of *Drosophila* co-expressing human amyloid precursor protein and BACE-1 specifically in the brain.

*Siolmatra brasiliensis* (Cogn.) Baill, (“taiuiá”, “cipó-tauá”) and its isolated substances (cayaponoside A1, cayaponoside B4, cayaponoside D, and siolmatroside I) were studied for relieving pain [15]. Hydroethanol extract, ethyl acetate fraction, and isolated saponins showed analgesic effects and reduced capsaicin- or glutamate-induced hypersensitivity by a mechanism involving muscarinic and opioid signaling.

Ethyl acetate, methanol, and aqueous extracts of aerial parts of *Anthemis tinctoria* var. *pallida* and *A. cretica* subsp. *tenuiloba* were investigated for their phenol and flavonoid content, antioxidant, and key enzyme (AChE, BChE. tyrosinase and α-glucosidase) inhibitory potentials [16]. Further, ex vivo studies highlighted neuroprotective properties after an excitotoxicstimulus promoting LDH level and 5-HT turnover normalization as well as the restoration of proteins involved in neuron morphology and neurotransmission.

Myint et al. [17] studied the activity of a methanol extract of *Smallanthus sonchifolius* leaf against a human hepatocellular carcinoma cell line. The extract reduced cell proliferation and cell migration, it also induced cell cycle arrest and necrosis in a concentration-dependent manner. Putative active components were melampolide-type sesquiterpenoids.

Antiproliferative properties were also depicted for the grape fruit essential oil [18]. Deng et al. detected by GC-MS twenty-four components (terpenes and oxygenated terpenes); the light phase oil displayed inhibitory effects on liver cancer cells proliferation, antimicrobial effects against *Bacillus subtilis*, *Escherichia coli*, *Staphylococcusaureus* and *Salmonella typhimurium* as well as antioxidant activity.

Antimicrobial activities were described for crude, phenolic-rich extracts of *Hibiscus sabdariffa Brassica oleracea* var. capitata f. rubra and *Beta vulgaris* [19]. Total anthocyanins, phenols, flavonoids contents were analyzed. Extracts and isolated compounds showed antimicrobial effects against pathogenic bacteria and fungi. Electron microscopy analysis revealed bacteria morphological alteration, indicating death and loss of cell content.

Pagano et al. [20] studied the non-edible outside layers of onion for wound healing. A hydroalcoholic extract was formulated in auto adhesive, biocompatible and pain-free hydrogel polymeric films, it showed antioxidant, radical scavenging, antibacterial and anti-inflammatory activities suggesting a potential dermal application for wound treatment.

Kurek-Gorecka and colleagues [21] reported the beneficial effects of bee products in dermatology and skin care. Honey, propolis, bee pollen, bee bread, royal jelly, beeswax and bee venom contain biologically active components, such as flavonoid schrysin, apigenin, kaempferol, quercetin, galangin, pinocembrin or naringenin. These components justify the use of bee products for medical or cosmetic skin treatment based on antibacterial, anti-inflammatory, antioxidant, disinfectant, antifungal and antiviral properties.

A protein fraction from *Ulva intestinalis* containing 51% of proteins and 22% of polysaccharides was analyzed and tested for the anti-aging potential, fibroblast proliferation and collagen and hyaluronic acid production on human fibroblast cell lines. A significant increase in collagen and hyaluronic acid production per cell, and a reduction in cell proliferation without increasing cell mortality were demonstrated [22].

UVB-induced skin damage in mice was reduced by dietary corn silk [23]. Oral administration decreased epidermal thickness, wrinkle formation, and positive staining for PCNA, Ki67, and 8-OHdG, and increased collagen staining. Pro-inflammatory NF-κB target genes and MMP-9 expressions were lowered, whereas TGF-β/Smad signaling increased. Low skin lipid peroxidation and blood DNA oxidation levels and high blood glutathione were detected in parallel with higher levels of catalase, SOD1 and glutaredoxin.

A wide description of *Ophiorrhiza rugosa* var. *prostrata* was performed by Adnan and coworkers [24]. The ethanolic extract of leaves, in three different vivo models, evoked antidiarrheal, anti-inflammatory, anthelmintic and antibacterial effects. Additionally, ADME and PASS analysis revealed a suitable profile for future medicinal development.

Almost 50 species of *Ophiorrhiza* plants were reviewed by Taher and colleagues [25]. The analysis revealed their wide distribution across Asia and the neighboring countries, whereby they were utilized as traditional medicine to treat various diseases. Biological activities encompass anti-cancer, antiviral, antimicrobial, and more. The genus propagation reported could produce a high quality and quantity of potent anticancer compound, namely camptothecin (CPT).

An updated snapshot of *Lamium* plants and their biological activities were provided by Salehi et al. [26]. Botanical, phytochemical and biological characteristics were described, highlighting antimicrobial, antiviral, anti-inflammatory, cytoprotective, anti-nociceptive properties.

Again, Salehi with an international team provided a deed analysis of the *Cucurbita* genus [27]. The traditional efficacy against gastrointestinal diseases and intestinal parasites were correlated with their nutritional and phytochemical composition. Among chemical constituents, carotenoids, tocopherols, phenols, terpenoids, saponins, sterols, fatty acids, and functional carbohydrates and polysaccharides were those occurring in higher abundance. More recently, a huge interest in a class of triterpenoids, cucurbitacins, has been stated.

A deep analysis of the *Fragaria* genus was presented by Fierascu and colleagues [28]. Strawberries possess biological properties, including antioxidant, antimicrobial and anti-inflammatory effects, but only a few species represent the subject of the last decade of scientific research. The main components identified in the *Fragaria* species were here described.

Zhang et al. [29] determined the processes and mechanisms of intestinal absorption of capilliposide B and C from *Lysimachia capillipes* Hemsl. Mechanisms involve processes such as facilitated passive diffusion, efflux transporters, and enzyme-mediated metabolism. Both capilliposides were suggested to be substrates of P-glycoprotein and multidrug resistance-associated protein 2. Capilliposide B may interact with the CYP3A4 system.

A phytochemical analysis on saccharide-containing compounds from *Eurycoma longifolia* was perfomed by Chua et al [30]. Non-toxic solvent fractionation increased the total saponin content, evoking anti-proliferative activity against human breast cancer cells.

Osthole was proposed for the treatment of tobacco mosaic virus [31]. Extracted from *Cnidium monnieri*, osthole showed comparable or stronger antiviral activity than eugenol and ningnanmycin. A direct effect on the viral particles was suggested.

A second edition of this Special Issue is in preparation.
